# Long-term survival of chronic dialysis patients following survival from an episode of multiple-organ failure

**DOI:** 10.1186/cc7867

**Published:** 2009-05-05

**Authors:** Richard J Chapman, Maie Templeton, Simon Ashworth, Robert Broomhead, Adam McLean, Stephen J Brett

**Affiliations:** 1 Department of Anaesthetics, Southampton University Hospitals NHS Trust, Anaesthetic Department, Mail Point 24, Southampton General Hospital, Tremona Road, Southampton, Hampshire SO16 6YD, UK; 2 Centre for Perioperative Medicine and Critical Care Research, Department of Anaesthetics and Intensive Care, Hammersmith Hospital, Imperial College Healthcare NHS Trust, Du Cane Road, London W12 0HS, UK; 3 Department of Renal Medicine, Hammersmith Hospital, Imperial College Healthcare NHS Trust, Du Cane Road, London W12 0HS, UK

## Abstract

**Introduction:**

This study aimed to examine the long-term outcome for patients with end-stage renal failure (ESRF) who survived multiple-organ failure.

**Methods:**

We performed a review of databases from the renal medicine service and intensive care units (ICU) of the participating hospitals within Imperial College Healthcare NHS Trust, London, UK. Patients with ESRF admitted to ICU who required support of two or more organ systems or were ventilated for more than 36 hours were included. To provide a comparison we examined the survival of a comparator group of ESRF patients in the general population with similar demographic and disease characteristics to our study group. We also examined the outcome for ESRF patients admitted to ICU who died prior to discharge.

**Results:**

Survival data for two years following discharge from ICU were examined for the impact of age, prior dialysis history, Acute Physiology and Chronic Health Evaluation (APACHE) II scores and medical or surgical status. Of the 199 patients who met the inclusion criteria, 111 (56%) survived their ICU stay. Sixty-two (56%) of the survivors remained alive two years following discharge. There was no group difference in survival with regards to age, dialysis history or APACHE II scores. Those admitted with a medical rather than surgical diagnosis were less likely to survive two years (*P *< 0.01). Patients who died in ICU had higher APACHE II scores (*P *< 0.0001) and were more likely to have a medical diagnosis. By log rank analysis two-year mortality was significantly higher (*P *= 0.003) in the ICU survivors than the comparator group with ESRF. This difference was lost when patients who died within a month of discharge were excluded.

**Conclusions:**

ESRF patients with multiple-organ failure have a high mortality, with the increased risk of death continuing into the early post-ICU period. Those with non-surgical diagnoses have the highest risk. Survival within the group who live beyond the early post-ICU period appears similar to the background population of ESRF patients.

## Introduction

The incidence and prevalence of end-stage renal failure (ESRF) is increasing, with an approximate doubling of patients requiring renal replacement therapy (RRT) per decade [1]. Recently published figures for the UK show a RRT incidence of 111 per million population (pmp) and a prevalence of 735 pmp [2]. Patients who require chronic renal dialysis carry a high burden of ill health and have an increased risk of death [1,3,4]. Morbidity is particularly associated with cardiovascular disease, with an increased incidence of myocardial infarction, cardiac failure and stroke due to the prevalence of hypertension, cardiac hypertrophy and ventricular dysfunction in this population [5-7]. Other health problems include sepsis, anaemia, bone disease, abnormalities of endocrine function (including diabetes mellitus), gastrointestinal complications, coagulopathies and disorders of the autonomic and peripheral nervous systems [7].

There have been few data published describing the effect of an episode of multiple-organ failure on the long-term survival of patients with dialysis-dependent chronic renal disease. Thus our primary objective was to examine the long-term survival of chronic dialysis patients who had survived an episode of multiple-organ failure, and to compare this with the survival of a group of chronic dialysis patients drawn from the background population. A secondary aim was to identify any relationship of age or prior chronic dialysis duration with subsequent survival.

## Materials and methods

As this study was an audit of historical data without intervention or patient involvement, the Chairman of the Institutional Review Board confirmed that formal ethical approval was not required.

### Setting

This was a retrospective study using the databases of the general intensive care unit (ICU) and renal unit of the participating hospitals (Hammersmith, Charing Cross and St. Mary's Hospitals, London). Patients included in the study were those with a chronic health diagnosis of dialysis-dependent (peritoneal or haemodialysis) ESRF who were admitted to the general adult ICU of the participating centres during the period 1999 to 2004, with a critical illness as defined below. The hospitals involved are tertiary referral hospitals, and the main centres for the regional renal medicine service (The West London Renal and Transplant Centre).

### Patients

For the purposes of this study critical illness was defined as admission to ICU and requirement for the support of two or more organ systems, and/or mechanical ventilation of more than 36 hours. By definition all patients required RRT, if admitted to the ICU for a long enough period. Support of one further organ system was therefore required for inclusion, although patients were eligible with one-organ failure if ventilated for 36 hours and then discharged from ICU before requiring dialysis. This definition was chosen to exclude those patients whose illness was not severe enough to require prolonged ICU care, for example those admitted after planned major surgery for a brief period of observation or mechanical ventilatory support. It was intended to include all other episodes of severe critical illness within the ESRF population.

### Sources of data

Data were collected from databases kept by the participating ICUs, and by the regional renal medicine service, which keeps a record of all patients with ESRF. Details extracted were age, sex, date of admission to both hospital and ICU, duration of mechanical ventilation, duration of multiple-organ support, medical or surgical status, elective or emergency status of surgery, acute physiology and chronic health evaluation (APACHE II) score, duration of dialysis history prior to admission, date of ICU discharge and date of hospital discharge or death if in hospital. For those patients remaining alive the regional renal database and hospital patient information systems were used to determine survival over a two-year period following each patient's discharge from the ICU. Mortality was recorded without further enquiry into cause, other than for those who died before hospital discharge.

A database of all patients who started on the chronic dialysis programme in London in 1997 was available for analysis. In order to provide a comparison, the survival of a cohort of patients (our comparator group) from this database was studied for two-year survival. These patients were selected to have a similar age (by excluding those with extremes of age, rather than detailed case-matching) and prior chronic dialysis duration to the study group; all patients in this group had started dialysis during 1997, and we started the survival analysis from the beginning of 2000. This ensured a median time on the dialysis programme equivalent to that of our main study population. There was no other discrimination or disease matching in selection of patients for the comparator group.

### Statistical analysis

The survival data were used to construct Kaplan-Meier survival curves for the two-year period following ICU discharge or from the start of 2000 for the comparator group. Patients were censored if transplanted or lost to follow up within this time period. Survival curve analysis was performed using the Log Rank test. For other data descriptive statistics were calculated, data checked for normality and subsequently student's t or Mann-Whitney U tests were used where appropriate. The effect of age and prior dialysis duration was examined using univariate analysis for their effect on survival to two years. The number of comparisons was modest and a significance level of 5% was selected. Statistical analysis was performed using Excel (Microsoft Corporation) and Prism (version 5, GraphPad Software, San Diego, CA, USA).

## Results

A total of 199 patients admitted to the ICU met our criteria for study inclusion. Of these, 111 (56%) were discharged alive from the ICU and were analysed as our survival cohort. Seventy-two (65%) of the survival cohort were male and 39 (35%) female. The mean length of ICU stay was 7.5 days (± 10.1), median five days (interquartile range = three to seven). Ninety-three patients (84%) received ventilatory support. Sixty-two patients from the survival cohort (56%) remained alive two years following discharge, and three (3%) received renal transplantation within the same time frame.

For the comparator group, 440 were alive at the start of 2000 and 254 were known to be alive at the end of 2001, with 32 patients receiving transplants and 21 being lost to follow up during this period (censored in the analysis); 134 patients had died during the study follow-up period.

**Figure 1:**
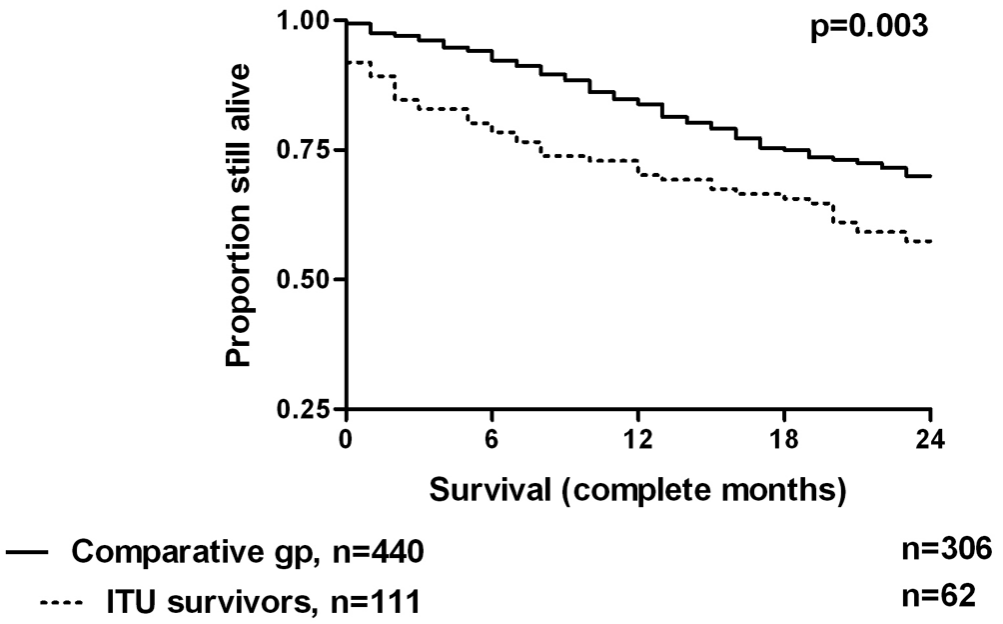
Kaplan-Meier survival curves for 111 dialysis-dependent patients discharged alive from intensive care unit and a comparative group of 440 dialysis-dependent patients who had not suffered a period of critical illness.

**Figure 2:**
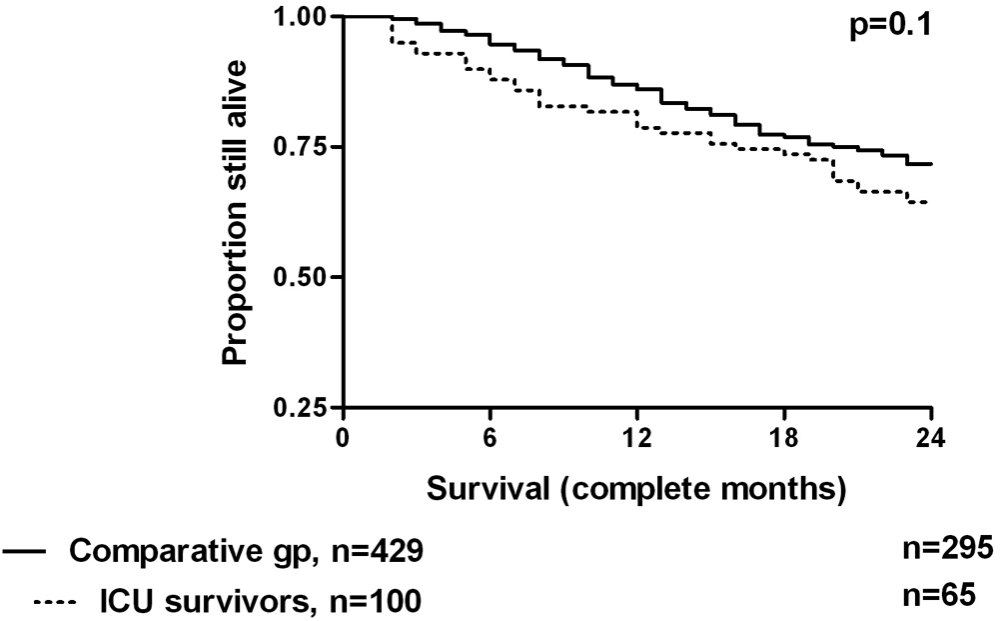
Kaplan-Meier survival curves for both cohorts with patients who died within one month removed from the analysis. ICU = intensive care unit.

The Kaplan-Meier survival curve is shown in Figure 1, and the effect of age and prior dialysis duration by outcome is shown in Table 1. There was a highly significant difference in survival between the two cohorts. Table 2 summarises the characteristics of the ICU cohort and the comparator group of chronic dialysis patients. Nine patients in the study group died within one month of discharge from ICU, all but one before hospital discharge. Visual inspection of Figure 1 suggests that the early deaths account for the difference in survival, so the analysis was re-run having removed patients from both cohorts who died within the first month of the two-year follow-up period (Figure 2). The difference between the curves is no longer significant.

**Table 1:** The effect of age and prior dialysis history on survival in ESRF patients discharged alive from ICU following an episode of critical illness

	Survived (n = 63)	Died (n = 48)	*P *value
Mean age (± SD)	58.2 (± 12.5)	60.1 (± 13.7)	0.6
Median days on dialysis prior to admission (IQR))	665 (182 to 2052)	1016 (296 to 2042)	0.37
Mean APACHE II score (± SD)	24.6 (± 5.6)	26.1 (± 5.7)	0.12

APACHE = Acute Physiology and Chronic Health Evaluation; ESRF = end-stage renal failure; ICU = intensive care unit; IQR = interquartile range; SD = standard deviation.

**Table 2:** Age and disease history characteristics of ESRF patients discharged alive from ICU following an episode of critical illness and a comparator group of ESRF patients in the general population

	Cohort group (n = 111)	Comparator group (n = 440)	*P *value
Mean age (± SD)	59.3 (± 13.0)	58.6 (± 13.5)	0.59 (t)
Days on dialysis prior to admission or inclusion (median (IQR))	744 (222–2042)	915 (816–995)	0.31

ESRF = end-stage renal failure; ICU = intensive care unit; IQR = interquartile range; SD = standard deviation.

**Figure 3:**
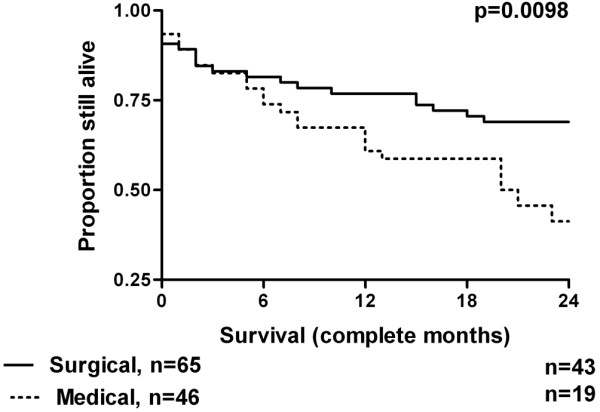
Kaplan-Meier survival curves of the intensive care unit survivors comparing medical or surgical status on admission.

**Figure 4:**
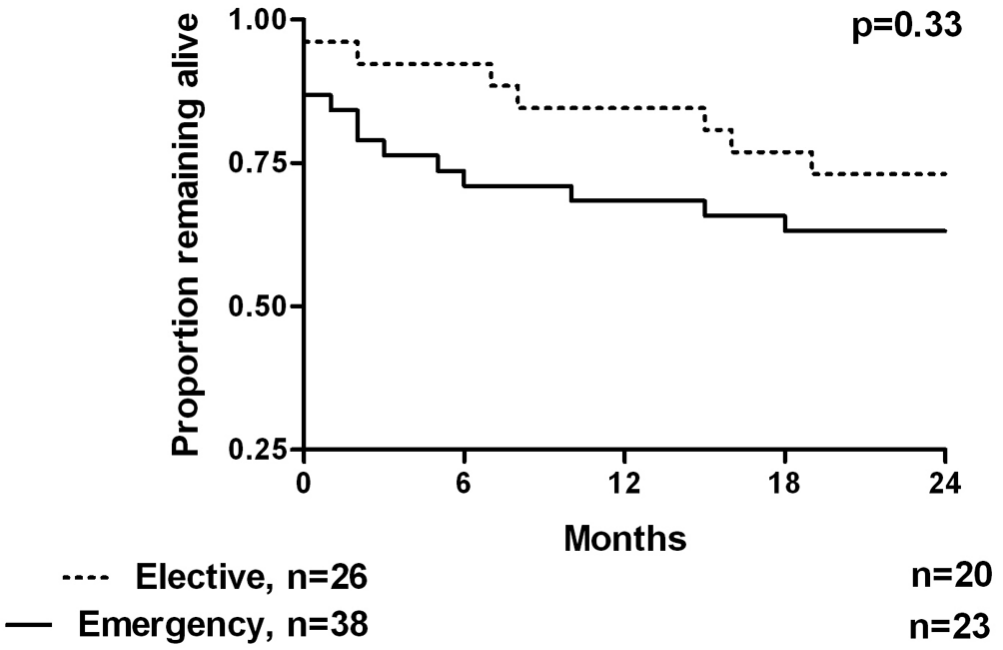
Kaplan-Meier survival curves of surgical intensive care unit survivors comparing emergency or elective status.

Long-term outcome was found to be significantly worse for patients whose reason for admission was not associated with surgery. Figure 3 demonstrates the survival curves for these two groups, with details summarised in Table 3. Initial survival appears identical, but the curves start to separate from around six months after ICU discharge. Figure 4 demonstrates the survival curves for surgical patients depending on emergency or elective status. Although emergency patients appear to have a worse survival profile, the numbers studied are too small to have shown a statistically significant difference.

**Table 3:** Age, disease history characteristics and APACHE II scores of ESRF patients discharged alive from ICU following an episode of critical illness, comparing those with a medical and surgical reason for admission

	Surgical (n = 64)	Medical (n = 47)	*P *value
Mean age (± SD)	59.6 (± 11.8)	58.9 (± 14.6)	0.75
Median days on dialysis prior to admission (IQR)	744 (292 to 1995)	790 (186 to 1936)	0.96
Mean APACHE II score (± SD)	24.4 (± 5.5)	26.2 (± 5.7)	0.12

APACHE = Acute Physiology and Chronic Health Evaluation; ESRF = end-stage renal failure; ICU = intensive care unit; IQR = interquartile range; SD = standard deviation.

Patients who died during their ICU admission (Table 4) had higher APACHE II scores, but were not significantly older and did not have significantly longer prior dialysis histories than those who survived to be discharged to the normal wards. Of these patients, only 15 had a surgical procedure associated with their final admission to ICU. Thus medical patients had a substantially greater chance of dying during their ICU stay, 61% versus 19% respectively (relative risk = 2.1, 95% confidence interval = 1.62 to 2.6, *P *< 0.0001).

**Table 4:** Age, disease history characteristics and APACHE II scores of ESRF patients with critical illness, comparing those who survived with those who died on the ICU

	ICU survivors (n = 111)	Died in ICU (n = 88)	*P *value
Mean age (± SD)	59.3 (± 12.9)	61.3 (± 13.6)	0.3
Median days on dialysis prior to admission (IQR)	744 (222 to 2042)	1139 (230 to 2028)	0.79
Mean APACHE II Score (± SD)	25 (± 5.7)	31 (± 8)	< 0.0001

APACHE = Acute Physiology and Chronic Health Evaluation; ESRF = end-stage renal failure; ICU = intensive care unit; IQR = interquartile range; SD = standard deviation.

## Discussion

In the UK, the ICU mortality for patients with multiple-organ failure is about 20 to 25% [8]. Our data suggest that chronic dialysis patients presenting with multiple-organ failure have, at 44%, a relatively high risk of dying during their acute illness in the ICU. However, for those who survive to hospital discharge, long-term survival is the same as for other chronic dialysis patients. Importantly, however, ICU survivorsoriginally admitted with non-surgical diagnoses have a worse long-term outcome, but this only becomes substantially apparent after discharge. Although detailed comparison of risk factors for the study and comparator groups has not been performed, the similarity of the long-term survival curves suggests that the background risks of the two groups are, indeed, comparable.

The unique features of this study are that the patients studied were included using a robust definition of critical illness, and that the duration of follow up was longer than any previously reported. We elected to stop the follow up after two years because arguably beyond this point the major factor determining mortality is underlying or novel serious disease, rather than the tail end of the index critical illness, although clearly this will not be true for everyone.

Long-term outcome studies have shown that patients discharged from the ICU demonstrate a mortality rate of 3.3 to 3.4 times the general population, although this returns to the expected level between two and four years after discharge [9,10]; intensive care mortality in these studies was 9.9 to 20.6%. Survival at five years was shown in the same studies to be 52.9 to 59.9%. In our study the ICU mortality was 44%, hospital mortality was 56% and survival at two years was 29%. However, the increased length of stay for our patients (7.4 ± 10.1 days compared with 3.3 ± 5.8 or 4.5 ± 7.2) and greater APACHE II scores (overall mean 27.6) suggests a sicker cohort of patients in our study compared with these general ICU populations.

As with previous studies we have demonstrated the importance of early death in producing increased mortality rates following ICU discharge. When death within one month (and almost exclusively in-hospital) are removed, the mortality rate for our patients appeared to be that expected for the background dialysis-dependent population. This effect of early deaths has previously been shown to be of greater importance in patients who are more unwell on admission to ICU [9], and the population with ESRF has demonstrably greater illness severity at admission than those without [11-13]. There are a number of possible explanations for the early deaths. None of the participating units discharge patients whose death is thought to be imminent. Of the 11 patients who died prior to hospital discharge, we determined that 10 had died with a decision either to withdraw active treatment or not to escalate treatment further due to severe burden of continuing ill health. Post-ICU mortality has been shown in general populations to be significantly higher in 'at-risk' patients following early discharge [14]. What our survival estimates do suggest is that ICU patients discharged alive from hospital have a survival prospect similar to the overall dialysis population.

Few published studies have looked at the impact of ESRF on survival post-ICU. Our observed ICU mortality is high compared with earlier studies that have shown mortality rates between 9% and 28.3% [11-13,15,16]. Clermont and colleagues [11] showed an ICU mortality of 11% for ESRF patients, two-times that of patients without renal failure. The mean length of stay for ESRF patients was five days, and they had significantly greater disease severity as measured by APACHE III scores than either those with acute renal failure (ARF) or no renal impairment. Despite higher APACHE III scores the hospital mortality for ESRF patients (14%) was lower than that for ARF patients who required dialysis (57%). The suggestion is that critical illness severe enough to result in ARF in those with previously normal kidneys would be associated with a particularly high mortality. In our study it is impossible to determine which patients would have developed ARF had they not already had ESRF, but the high mortality demonstrated implies that our definition of critical illness is in the order of this severity. An important caveat is that ICU mortality will, to some extent, reflect the referral practice of the individual nephrology services, thus limiting how far ICU mortality statistics may be generalised.

Manhes and colleagues [12] studied an ESRF patient group whose age (62.8 ± 14.6 years) and length of ICU stay (6.2 ± 9.9 days) were similar to that in our study. They demonstrated an ICU and hospital mortality of 28.3% and 38%, respectively, and confirmed the increased illness severity and mortality conferred by ESRF compared with non-dialysed patients. Survival at six months was 52.2%.

Hutchison and colleagues [13] found that 1.3% of all ICU admissions (for ICUs participating in the Intensive Care National Audit & Research Centre (ICNARC) case mix programme in England, Wales and Northern Ireland) were for patients receiving chronic dialysis. These patients tended to be younger and more often male than those without ESRF. Their APACHE II scores when compared with non-ESRF patients again demonstrated an increased illness severity (24.7 v 16.6), but the mean length of stay (1.9 days) is much less than that seen in our study. Their observed hospital mortality was 45.3% for patients with ESRF (31.2% for those without). As in our study, non-surgical reason for admission and emergency surgery were associated with an increased mortality. This report did not use the same definition of multiple-organ failure as used for enrolment in our study.

The cohort of ESRF patients in the study by Uchino and colleagues had a mean APACHE II score of 21.8, an ICU mortality of 22% and a hospital mortality of 34% [15]. In this study the observed mortality for patients with ESRF was similar to that of patients developing ARF. They calculated that 2% of patients with ESRF would require admission to ICU each year, a figure confirmed by a more recent study in which 20% of dialysis-dependent patients needed ICU admission over a period of nearly six years [16]. In this latter study the observed ICU mortality was just 9% despite 76% of ESRF patients being admitted for medical rather than surgical reasons. The contrast to the figures seen in our study is explained by the absence of non-renal organ failure patients in this group, with 48% requiring no other organ system support.

Bell and colleagues recently published a meticulous study of short and long-term outcome for ESRF patients treated in the ICU [17]. They demonstrated a 90-day mortality of 42% for all patients, with a higher mortality shown in those with co-morbidities of diabetes and heart disease. In contrast to our study they found that long-term risk of death remained elevated in the ICU survivors compared with the background population of ESRF patients. However, their study cohort included all patients admitted to ICU. In our study, long-term parity of cohort and comparator groups was seen when early deaths were excluded, and we did not include patients who died during their ICU admission.

We have been careful to avoid using the term 'control' for our comparator group. This group represented retrospective data and was matched *a priori *for approximate age and prior duration of chronic dialysis before the analysis was run. There was no other disease matching. Importantly, we have no information on the final illnesses of those who died, which may have included periods in the ICU. We elected not to exclude such patients as we used the comparator group to estimate the background mortality of chronic dialysis patients, and to exclude intensive care from this would be fallacious. As such, there is the possibility of overlap between the comparator group and our study cohort, which may have lessened the observed differences. A total of seven patients from our survivor cohort were also included in the comparator group, representing fewer than 2% of the latter. We thus expect the magnitude of any effect on comparisons to be small.

We recognise that the lack of observed difference for the comparison between our study group (early deaths excluded) and the comparator group may be due to the small size of our sample. For this observation the relative risk was calculated as 0.9, and the power was 0.13. This risk of a type II error will have been increased by the small degree of overlap between our groups. However, a difference in mortality, if present, would arguably be very small. Based on our observations, a prospective study with an allocation of 1:1 for ICU survivors and controls, excluding early deaths, to demonstrate a mortality difference with a relative risk of 0.9 and a study power of 0.8 would require enrolment of 2150 subjects.

For a comparison of this type, mortality is a surprisingly complex endpoint. In future studies, there is an argument that composite endpoints, now commonly used in cardiovascular intervention trials, should also be used for analysis. These might include defining subjects as being free from death and either ICU admission or additional organ failures, as ICU admission criteria are extremely variable.

We also recognise the lack of data on co-morbidities for our study population. Information available from the early ICU databases was not sufficient to allow full collection of data on enough variables for a meaningful multivariate analysis. It has been shown that co-morbidities such as diabetes and heart failure have an important impact on survival in this cohort of patients. The very high mortality in the group with a medical rather than surgical reason for admission may have been influenced by a high incidence of co-morbidities such as these. We recognise that further investigation of these variables would be a desirable aim for a larger, prospective study.

Although the patients who died in ICU had higher APACHE scores and were proportionately less likely to be surgical, taken overall we have been unable to identify any systematic patient factors – age, APACHE II score or prior duration of chronic renal failure – which were so strongly associated with worse outcomes that they might be used to support decisions not to admit patients to intensive care. Having said that, one striking observation is that of the 120 'medical' patients originally admitted to intensive care, only 46 were discharge from ICU alive and only 19 remained alive two years after discharge.

## Conclusions

Our study has demonstrated a high mortality rate for patients with ESRF admitted to ICU with multi-organ failure. For survivors there appears to be a worse long-term outcome than for a general ESRF population. This excess mortality is, however, caused by the high number of deaths in the first month following discharge from ICU, largely occurring while still in hospital. The suggestion is that patients remain at high risk in the early stages following ICU discharge. Those patients admitted for non-surgical diagnoses are particularly at risk, and this high risk appears to continue.

Although the long-term survival rates return to that of the background population with ESRF, this study has not allowed any determination of the effect of ICU admission on quality of life for these patients; this remains an important question.

The ICU and hospital mortality rates in our study are high in comparison to previous studies of ESRF patients. Our inclusion criteria have selected a group whose diagnosis of multiple-organ failure indicates a very high level of illness severity for which such high mortality rates would not be unexpected.

Future prospective studies should focus on patients' disease characteristics and co-morbidities in order to determine which groups of ESRF patients are potentially the most and least likely to benefit from ICU admission. Strategies should be in place to accommodate any overlap between study subjects and the comparison population. The degree of any such overlap would be a reflection of local ICU referral practice.

## Key messages

• Patients with ESRF and a critical illness have a high mortality rate in the ICU.

• Mortality in this group remained high compared with the background population of patients with ESRF for the duration of our study (up to two years following ICU discharge).

• Following discharge from the ICU, risk of death is highest in the first month. Longer-term, the mortality rate appears to approximate that of comparable ESRF patients from the background population.

• Patients admitted to the ICU with a non-surgical diagnosis have a higher risk of death compared with surgical patients. This difference remained both in the ICU and post-discharge for the duration of our study.

## Abbreviations

APACHE: Acute Physiology and Chronic Health Evaluation; ARF: acute renal failure; ESRF: end-stage renal failure; ICNARC: Intensive Care National Audit & Research Centre; ICU: intensive care unit; pmp: per million population; RRT: renal replacement therapy.

## Competing interests

The authors declare that they have no competing interests.

## Authors' contributions

RC acquired, correlated and processed data, helped with study design and drafted the manuscript. MT acquired data. SA acquired data. RB acquired data. AM acquired and processed data and helped with study design. SB conceived of the study, participated in its design and coordination, performed statistical analysis and helped to draft the manuscript. All authors read and approved the manuscript.

## Authors' information

RC was a clinical fellow at Imperial College Healthcare NHS Trust at the time of study design, data collection and analysis.
